# Management of Short Bowel Syndrome With Chronic Intestinal Failure: A Single-Center Experience in Portugal

**DOI:** 10.7759/cureus.63443

**Published:** 2024-06-29

**Authors:** Marisa D Santos, Vania Magalhães, Luis Loureiro, Pedro Pina, Ana Castro, Paulina Aguiar, Anabela Rocha

**Affiliations:** 1 Colorectal Surgery, Instituto de Ciências Biomédicas Abel Salazar (ICBAS), University of Porto, Porto, PRT; 2 Colorectal Surgery, Unidade Local de Saúde de Santo António (ULSSA), Porto, PRT; 3 Nutrition, Unidade Local de Saúde de Santo António (ULSSA), Porto, PRT; 4 Vascular Surgery, Unidade Local de Saúde de Santo António (ULSSA), Porto, PRT; 5 Anesthesiology, Unidade Local de Saúde de Santo António (ULSSA), Porto, PRT; 6 Nephrology, Unidade Local de Saúde de Santo António (ULSSA), Porto, PRT; 7 Pharmacy, Unidade Local de Saúde de Santo António (ULSSA), Porto, PRT

**Keywords:** complications of parenteral support, teduglutide, home parenteral nutrition, chronic intestinal failure, short bowel syndrome

## Abstract

Introduction: Short bowel syndrome with chronic intestinal failure (SBS/CIF) is the inability to maintain protein-energy, fluid, electrolyte, or micronutrient balance due to a short bowel. Although SBS/CIF is rare, its clinical management is complex, challenging, expensive, and time-consuming.

Aim: This study aimed to analyze a single center's experience with SBS/CIF in adult patients treated with home parenteral nutrition (HPN).

Materials and methods: A retrospective single-center analysis of all 13 consecutive adult patients with SBS/CIF was included in an HPN program between January 1994 and August 2023.

Results: Between 1992 and 2023, 13 patients were included in an HPN program. The primary underlying pathology was acute mesenteric ischemia. The median age of starting HPN was 44 years. Most were subjected to several surgeries of extensive intestinal resection with posterior intestinal reconstruction. Five of the 13 patients died while on HPN with a median duration of 42 months. The causes of death related to HPN were catheter sepsis, endocarditis with cardiac failure, or hepatic failure. One patient died due to underlying pathology: pelvic abscesses and bleeding related to radiotherapy. Eight patients remain alive, with a median time of HPN of 173 months. During the HPN support, the most frequent complications were venous catheter infection and venous territory thrombosis. None of the eight patients alive have hepatic failure. Two patients recently started teduglutide with good tolerance and need a reduction in HPN support. All eight patients have a satisfactory quality of life (parenteral support needs range between five and two nutrition bags per week).

Conclusion: Home parenteral nutrition remains the gold standard of SBS/CIF treatment, although teduglutide may reduce HPN needs and complications and provide a better quality of life. Despite the small number of patients, the results shown in this study are not inferior to those in large-volume centers. The existence of the commitment and interest of professionals involved in SBS/CIF at Centro Hospitalar Universitário de Santo António, Portugal, was a fundamental key to achieving those results. A multidisciplinary healthcare group for HPN support can be essential to ensuring these patients' survival and quality of life.

## Introduction

Intestinal failure is characterized by the inability to maintain adequate nutrition via the intestine. It can be subdivided into three groups: acute, usually self-limiting, of short duration and appearing in the postoperative period; subacute, for patients who are metabolically unstable, usually septic, and need prolonged artificial nutrition; and chronic, which requires long-term parenteral nutrition or intravenous (IVS) to maintain health and/or growth, appearing in metabolically stable patients who are incapable of maintaining adequate absorption of macronutrients and/or water and electrolytes through the intestine [1, 2].

Short bowel syndrome (SBS) is a subgroup of chronic intestinal failure, a rare entity usually resulting from extensive intestinal resections requiring prolonged parenteral nutrition as the primary therapy, ideally performed at home [3]. Home parenteral nutrition (HPN) means the patient does it at home through a central access (long-term central catheter), a nutritional bag, and/or a fluids and electrolytes bag. Home parenteral nutrition, the basis for treating SBS, is not free of serious complications, such as infection and thrombosis of central venous accesses, metabolic disturbances, and liver disease. The mortality risk in these cases can reach 10% to 15% when this type of nutrition lasts more than two years [4]. Teduglutide, an analog of glucagon-like peptide-2 (GLP-2), helps to reduce the need for parenteral support (PS) in patients with SBS with chronic intestinal failure (SBS/CIF). In managing SBS/CIF, teduglutide can also decrease complications associated with HPN, leading to a better quality of life and improved survival. It's important to note that the teduglutide response varied in timing and magnitude due to different characteristics of SBS/CIF, such as the cause of major intestinal resection, remnant bowel anatomy, the presence or absence of a stoma, and PS volume requirements. The guidelines for HPN are well established and help in correcting SBD/CIF patient orientation [5, 6].

In Portugal, the experience with SBS/CIF failure in adult patients in hospital centers nationwide is minimal. Centro Hospitalar Universitário de Santo António in Porto is the only national public institution that has maintained an HPN program for these types of patients over the last 30 years [7].

Given that SBS/CIF is a rare condition and difficult to handle, a reflection on this entity and the best way to treat it is the purpose of this article, based on the experience gained.

## Materials and methods

A retrospective single-center analysis of all consecutive adult patients with SBS/CIF on HPN between January 1994 and August 2023 was conducted. Primary outcomes were patient survival; secondary outcomes were the main difficulties in the HPN implementation, complications related to HPN (such as catheter sepsis and catheter thrombosis, fluid and electrolyte abnormalities, glucose intolerance, renal disease, liver disease, osteopathy, fatigue, and diarrhea), and quality of life.

Inclusion criteria

Adult patients with SBS/CIF treated in the last 30 years included in an HPN program at Centro Hospitalar Universitário de Santo António were a part of the study.

Exclusion criteria

Patients with sub-acute intestinal failure evolved favorably without needing home PS, and those who evolved unfavorably died without the opportunity to be included in this program were excluded.

Patients

The characteristics of the patients are described in Table 1. 

**Table 1:** Patient characteristics (age, sex, underlying pathology, remnant bowel) and SBS therapy details (type and duration) ♀: female; ♂: male; †: deceased CIF: chronic intestinal failure; HPN: home parenteral nutrition; GLP- 2: glucagon-like peptide-2; SBS: short bowel syndrome

Patient ID	Sex	Age at CIF diagnosis	HPN year	HPN duration (months)	Etiology	Remnant bowel	Intestinal stoma	GLP-2 analog therapy
1	♀	33	1994	68 ^†^	Mesenteric ischemia	Duodenum, colon, rectum	No	No
2	♂	35	1996	320	Mesenteric ischemia	Jejunum (15 cm), colon, rectum	No	No
3	♂	34	1996	27 ^†^	Mesenteric ischemia	Jejunum (fistula), ileum (3 cm), rectum	No	No
4	♀	47	2002	21	Mesenteric ischemia	Jejunoileum (18 cm), ICV, colon, rectum	No	No
5	♀	48	2003	13 ^†^	Radiation enteritis with intestinal ischemia	Jejunum (100 cm), sigmoid, rectum	No	No
6	♀	38	2007	248	Mesenteric ischemia	Jejunum (5 cm), left colon, rectum	No	No
7	♂	39	2009	188	Intestinal aganglionosis	Jejunoileum (ileostomy)	Yes	No
8	♀	32	2009	163	Intestinal volvulus with intestinal ischemia	Duodenum, left colon, rectum	No	No
9	♀	46	2009	163	Mesenteric ischemia	Jejunum (50 cm), left colon, rectum	No	Yes
10	♀	51	2010	87 ^†^	Radiation enteritis with intestinal ischemia	Jejunoileum (40 cm), left colon (colostomy)	Yes	No
11	♂	53	2013	16 ^†^	Mesenteric ischemia	Jejunum (50 cm), left colon, rectum	No	No
12	♂	48	2015	79	Mesenteric ischemia	Jejunum (50 cm), ileum (10 cm), ICV, left colon, rectum	No	Yes
13	♀	59	2019	43	Mesenteric desmoid tumor with Intestinal ischemia	Jejunum (10 cm), transverse and left colon, rectum	No	No

The primary underlying pathology was acute mesenteric ischemia. All patients underwent several surgeries for intestinal reconstruction. In most patients, the small intestine remained between 10 and 60 cm (jejunum or ilion), with the left colon preserved. The intestinal extent varied between patients; the smallest consisted only of the duodenum and colon (patients one and eight), and the largest consisted of 60 cm of jejunum plus 10 cm of ileum and the left colon (patient 12). Only two patients had the ileocecal valve preserved (patients four and 12). Most cases resulted from intestinal vascular ischemia, but we also had a case of volvulus, a case of desmoid tumor, a case of radiation enteritis, and one case of intestinal dysmotility. Of the patients in the program, one had an ileostomy due to intestinal aganglionosis, one had an ileostomy and a high-output jejunum fistula, and one had a colostomy; all others had colon continuity.

The first patient with CIF in an innovative program in Portugal emerged in 1994 (a case report was previously published). The patient remained in that condition for five years and died of an intravascular catheter-related bloodstream infection (CRBSI). The third patient emerged in 1996, and he is still in this program, accompanied by other cases that have appeared over this time. The incidence of CIF in our hospital has been decreasing. Overall, 13 adult patients were included in the HPN program from the beginning, of which eight are currently alive; 62.5% are female and the age at CIF diagnosis was 43.7 years old. The median age of the starting HPN was 44 years. At the date of writing this manuscript, the average age of participants was 58.4 years (minimum 48 and maximum 68 years). Table 2 provides a summary of the patient's underlying health conditions (comorbidities) and the probable cause of death at the time it occurred.

**Table 2:** Patients' comorbidities and cause of death † - deceased; *CRBSI: catheter-related bloodstream infections; COPD: chronic obstructive pulmonary disease

Patient ID	Comorbidities	Death	Cause of death
1		Yes	*CRBSI
2	Severe osteoporosis; cerebrovascular disease, heart disease; atrial fibrillation	No	
3		Yes	Hepatic cirrhosis with portal hypertension
4		No	
5	Radiation enteritis	Yes	Kidney failure, heart failure
6	Chronic kidney disease; renal lithiasis, right nephrectomy; recurrent urinary infections with bacteremia and pancytopenia. osteoporosis; arterial hypertension; depressive syndrome	No	
7	Neuropathic bladder; supra-pubic cystotomy; thrombosis of the superior vena cava from the right jugular veins; pseudo-epileptic seizures; past drug addiction, active smoking	No	
8	Metabolic bone disease with chronic polyarthritis of the small joints; paricalcitol-induced adynamic bone disease	No	
9	Essential tremor	No	
10	Rectal neoplasm; ovarian neoplasm; radiation enteritis; enterovesical fistula; recurrent urinary tract infections - pyonephrosis – nephrectomy; nephrostomies	Yes	Urinary infection, hepatic failure
11	Heart failure, COPD, diabetes, arterial hypertension, diabetic nephropathy stage IV, peripheral vascular failure, metabolic syndrome, paroxysmal atrial fibrillation, cardiorespiratory failure	Yes	Bacterial endocarditis, heart failure, cardiorenal syndrome type 2
12	Dysfibrinogenemia; stage G2/A3 chronic renal disease; post-cholecystectomy status (Sep/2018); diverticular disease; sigmoidectomy in Mar/2020; bifascicular block; history of acute pulmonary embolism; pulmonary tuberculosis; smoker; hyperuricemia with a history of gouty arthritis – controlled without medication; severe allergic reactions to multiple drugs tunnel syndrome	No	
13	Cholecystectomy; hysterectomy; chronic gastritis; Meniere syndrome; bilateral carpal tunnel syndrome	No	

Patient education and training program for HPN administration 

During the hospitalization, the patients learned how to administer HPN and recognize the main alarm signs when going to the hospital. Eleven out of the 13 patients achieved autonomy in managing their parenteral nutrition at home, which included self-management of the central venous catheter (CVC). The other two have problems with learning, and parenteral nutrition is made with nursing help. 

Central venous access and nutritional support

The patients usually have a long-term catheter, a tunneled one, placed in the right subclavian vein for parenteral nutrition administration. The catheter is flushed with heparin after IV administration of parenteral nutrition. We don´t routinely use taurolidine 4% at each catheter disconnection. 

Patients are independent in preparing the nutrient bags and undergo a cyclical infusion, on average, for 10 hours at night. All patients supplement the standard bag with water-soluble vitamins and fat-soluble and trace elements in each parenteral nutrition administration. Some patients need to supplement electrolytes in addition to those present in the nutritional admixture, particularly magnesium.

Apart from one patient who has recently started personalized parenteral nutrition, all others use standardized tri-compartmentalized bags. 

Teduglutide therapy

Our hospital only authorized the use of teduglutide therapy for adults in 2019. Out of eight patients, only two (patients nine and 12) met the health and socio-intellectual conditions criteria for utilizing the treatment. We follow the criteria described by Pironi et al. [5]. The patients started this therapy in 2022. The dose was adjusted to the patient‘s weight and administered once a day subcutaneously. The side effects of teduglutide are rare and easily resolved. The main difficulty could be adjusting bag volume during the weeks after the teduglutide introduction in patients with heart failure. In those cases, metabolic dysfunction and the risk of acute pulmonary edema are real. In our two patients, the volume bag reduction and the adjustment of the current parenteral nutritional supply were made progressively eight weeks after teduglutide started, taking into account weight, body composition, symptoms, and the analysis of the patient. 

Culinary food support

Only patient seven has an oral intake limited to liquid foods, and all others follow family eating routines.

Follow-up

These patients are regularly followed up with a multidisciplinary approach, including nutrition appointments. In each consultation, the nutritional status, including weight, body mass index (BMI), and whole-body composition, is assessed using bioelectrical impedance (Quantum II Body Composition Analyser, RJL Systems, Santeramo in Colle, Italy). Eating and bowel habits are evaluated with a high degree of detail. Food consumption is assessed using the 24-hour-recall method and a food frequency questionnaire, although patients fill out food diaries whenever clinically necessary. 

Blood tests with a sizeable nutritional profile regularly follow the updated European Society for Clinical Nutrition and Metabolism (ESPEN) guidelines on long-term HPN [8]. These tests allow the monitoring of adherence and therapeutic adequacy. Nutritional interventions are based on the results of all these assessments.

Various medical exams are conducted routinely to assess the health of the liver, kidneys, and other organs. These exams include abdominal ultrasound, bone scintigraphy, and upper and lower-case complications related to endoscopy. In case of complications related or unrelated to HPN, additional tests such as chest and abdominal CT scans, liver MRIs, or capsule endoscopies may also be performed. For example, the liver MRI was performed when patients presented changes in liver function, and the capsule endoscopic was performed on the patients for whom we needed to discard small intestine pathology before starting teduglutide.

In the event of hospitalization, patients are evaluated closely by the same team that follows them in an outpatient clinic.

## Results

Nutritional support

Patients in the HPN program administer parenteral nutrition on average 4.9 days a week (minimum: two and maximum: seven). The mean daily volume administered is 922 mL (minimum: 282 and maximum: 1,500 mL), corresponding to 6,452 mL per week (minimum: 1,972 and maximum: 10,500 mL). The mean daily energy intake is 986 kcal, 120 g of carbohydrates, 39 g of protein, and 35 g of lipids. All patients supplement the standard bag with water-soluble vitamins and fat-soluble and trace elements in each parenteral nutrition administration. Some patients need to supplement electrolytes in addition to those present in the nutritional admixture, particularly magnesium. Table 3 presents the different needs and characteristics of nutritional bags and supplies between genders.

**Table 3:** Current parenteral nutritional supply of the cohort of patients with chronic intestinal failure in a home parenteral nutrition program managed by the Colorectal Surgery Unit. ♀: female; ♂: male

		Weekly administration				Daily estimation			
	Sex (n)	Mean	SD	Minimum	Maximum	Mean	SD	Minimum	Maximum
Volume (mL)	♀ (n=5)	6134	3272.0	1972	10500	876	467.4	282	1500
	♂ (n=3)	6982	3029.5	3944	10003	997	432.8	563	1429
Energy (kcal)	♀ (n=5)	6285	2793.5	2200	8670	898	399.1	314	1239
	♂ (n=3)	7927	3500.3	4400	11400	1132	500.0	629	1629
Glucose (g)	♀ (n=5)	765	347.1	250	1050	109	49.6	36	150
	♂ (n=3)	960	450.3	500	1400	137	64.3	71	200
Aminoacids (g)	♀ (n=5)	246	102.5	100	328	35	14.6	14	47
	♂ (n=3)	315	119.0	200	438	45	17.0	29	63
Lipids (g)	♀ (n=5)	220	104,8	76	345	31	15,0	11	49
	♂ (n=3)	277	124.0	152	400	40	17.7	22	57
Sodium (mmol)	♀ (n=5)	310	214.7	80	543	44	30.7	11	78
	♂ (n=3)	345	182,6	160	525	49	26.1	23	75
Potassium (mmol)	♀ (n=5)	187	82.9	60	280	27	11.8	9	40
	♂ (n=3)	210	90.0	120	300	30	12.9	17	43
Magnesium (mmol)	♀ (n=5)	93	102.6	10	252	13	14.7	1	36
	♂ (n=3)	48	41.8	20	96	7	6.0	3	14
Calcium (mmol)	♀ (n=5)	45	38.9	5	66	6	5.6	1	9.4
	♂ (n=3)	30	23.7	10	56	4	3.4	1	8
Phosphate (mmol)	♀ (n=5)	137	101.4	24	260	20	14.5	3	37
	♂ (n=3)	155	93.8	48	224	22	13.4	7	32
Chloride (mmol)	♀ (n=5)	267	125.9	70	386	38	18.0	10	55
	♂ (n=3)	303	156.9	140	453	43	22.4	20	65
Zinc (mmol)	♀ (n=5)	0.376	0.40	0.08	1.05	0.05	0.058	0.01	0,15
	♂ (n=3)	0.753	0.51	0.16	1.05	0.11	0.073	0.02	0.15
Acetate (mmol)	♀ (n=5)	272	72.1	180	335	39	10.3	26	48
	♂ (n=3)	389	64.5	315	435	56	9.2	45	62

Apart from one patient who recently started individualized parenteral nutrition (compounded PN), all others use standardized, commercially available multi-chamber bags. The need for a particular formulation tailored to the patient arose due to a chronic renal disease that required specific water volumes and a lower load of amino acids, potassium, and phosphorus. Six of the eight patients completed autonomous in-bag nutrition manipulation. All eight know the warning signs of CVC infection and signs of metabolic dysfunction. They can call staff or go to the hospital emergency room if they have doubts. They have preferential service. In that way, the clinical problems are minimized.

Teduglutide therapy

Two (patients nine and 12) of the eight recently started teduglutide with good tolerance and reduction needs for HPN support. They aren’t showing any side effects, despite the comorbidities presented by patient 12. One of the patients has already reduced parenteral energy intake by 50%. After six months, teduglutide treatment significantly reduced PN days, caloric needs, infusion time, and infusion volume. The other had an infectious intercurrence (pleural tuberculosis) with prolonged hospitalization and changes in nutrition, which influenced the result, maintaining the initial intake today. It should be noted that the patient who reduced her intake maintained her weight and body composition.

Nutritional status

Table 4 contains a description of the anthropometric assessment of HPN patients.

**Table 4:** Current nutritional status of the cohort of patients with chronic intestinal failure in a home parenteral nutrition program managed by the Colorectal Surgery Unit. ♀: female; ♂: male

	Sex	Mean ± SD	Minimum	Maximum
Body mass index (kg/m^2^)	♀ (n=5)	23.6 ± 3.1	18.4	26.8
	♂ (n=3)	21.5 ± 5.34	18.3	27.7
Fat mass (%)	♀ (n=5)	31.5 ± 9.82	14.5	39.5
	♂ (n=3)	13.9 ± 6.41	6.9	19.5
Muscle mass (kg)	♀ (n=5)	22.6 ± 3.25	18.8	27.2
	♂ (n=3)	33.4 ± 8.88	23.6	40.9
Basal metabolic rate (kcal)	♀ (n=5)	1199 ± 99.6	1121	1370
	♂ (n=3)	1650 ± 216.1	1401	1776
Phase angle (º)	♀ (n=5)	5.0 ± 0.18	4.8	5.2
	♂ (n=3)	5.7 ± 1.51	4.2	7.2

Data from the last evaluation of each patient show a mean BMI of 22.8 kg/m^2^ (two patients at the upper limit of the malnutrition WHO classification and three at the lower limit of the overweight classification; data not shown). Phase angle was 5.0 in females and 5.7 in males, only below the limit in patient 13, possibly because she is still in an adaptation phase. A mean fat mass of 13.9% was obtained for the male sex (below the limit established by the bioimpedance equipment) and 32.0% for the female sex (above the limit established by the bioimpedance equipment). Regarding muscle mass, male sex patients presented a mean of 33.4 kg compared to 22.0 kg for female sex patients (Table 4).

Complications related and unrelated to HPN

Five of the 13 patients were deceased. These five deceased patients were on HPN for a median duration of 42 months (87-13 months). The causes of death related to HPN were catheter sepsis (two patients), endocarditis and cardiac failure (one patient), and hepatic failure (one patient); the causes of death unrelated to HPN were necrotic tissue, sepsis, pelvic abscesses, and pelvic bleeding due to radiation enteritis (one patient). 

Eight patients remain alive, with a median HPN of 176 months (324-48 months). Three of the eight have health comorbidities, making the HPN's management difficult.

During the HPN support in these eight patients, the main complications related to HPN were venous catheter infection and venous territory thrombosis. The incidence rate of central line-associated bloodstream infections (CLABSIs) was 0.46 infections per 1,000 catheter days. The frequency of the CVC infection is not the same in all patients. One of the patients kept the same catheter without complications for more than eight years. Catheter changes occur due to material deterioration. Urosepsis and metabolic dysfunction were the second-most frequent problems. None of the eight patients alive have hepatic failure. 

**Figure 1:**
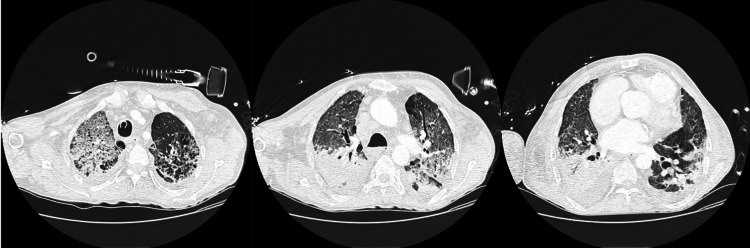
Lung infection due to COVID-19 in patient two

In these eight patients, the main complications unrelated to HPN were an auricular flutter with cardiac insufficiency, urosepsis with chronic renal dysfunction, acute cholecystitis, tuberculosis, and COVID-19 infection. The infection of COVID-19 in those patients was a severe problem during the pandemic. Four of our eight patients had a COVID-19 infection. One of them had a severe disease with intensive care hospitalization: lung infection (Figure 1) and posterior thrombosis of the central venous territory. 

**Figure 2:**
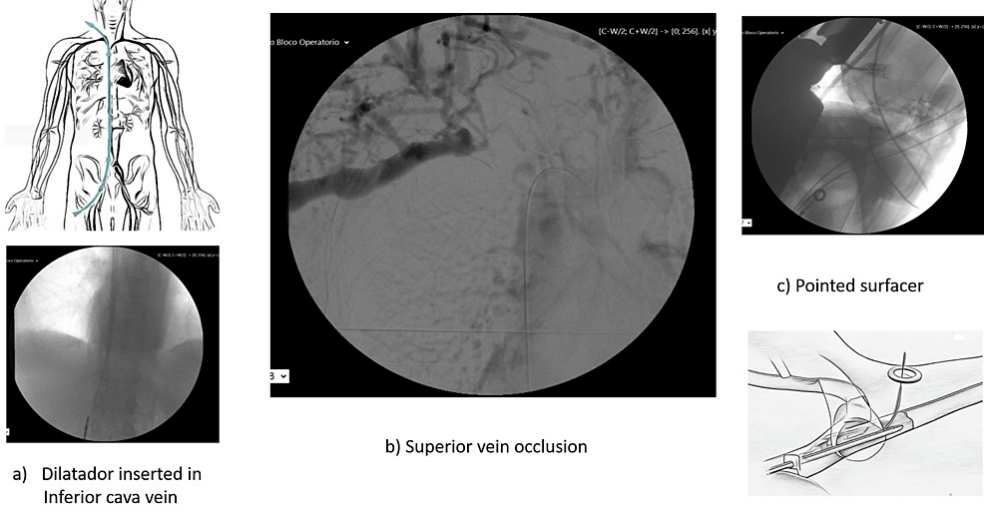
New right thoracic venous access after vein recanalization with the surfacer inside-out system. Procedure realized in patient two. Image adapted from https://www.merit.com/now-available-in-the-us-surfacer-system-provides-treatment-option-for-venous-obstructions/ and https://vascularnews.com/bluegrass-vascular-announces-ce-mark-approval-and-successful-commercial-use-of-surfacer-inside-out-access-catheter-system/ Graphical images created on Procreate (Savage Software Group Pty Ltd, Hobart, Australia)

The problem was fixed by introducing a thoracic venous catheter using the surfacer inside-out technique (Figure 2). 

Quality of life

All eight patients have a satisfactory quality of life, and their need for parenteral support varies between five and two nutrition bags per week. Six patients receive parenteral nutrition at home at night, while the remaining two receive it twice a week in a day hospital for four to six hours. These nutritional schedules enable patients to lead a lifestyle similar to that of the general population.

None of the patients practice scheduled and regular physical activity; they only do activities of daily living.

Regarding occupation, although they are very active in their daily lives, two patients work for their own companies. Another patient visits her daughter, who lives in another European country and travels by plane.

Seven are married, and the majority are in stable families. One lives alone. None need active psychiatry or psychological support.

## Discussion

Short bowel syndrome with chronic intestinal failure is the inability to maintain protein-energy, fluid, electrolyte, or micronutrient balance due to a short bowel [3]. 

In Portugal, extensive adult intestinal resections that lead to SBS are generally performed in urgent situations due to acute mesenteric ischemia. Other pathological entities may require extensive intestinal resections performed on an elective or urgent basis, such as Crohn's disease [8-10], radiation enteritis [11], and severe complications of digestive, severe gastrointestinal dysmotility disorders [12], digestive surgical complications, or intestinal bypass preparations due to oncological disease [13], which can also result in SBS. Our experience is minimal in those cases; we haven’t experienced oncologic intestinal obstruction patients. 

We have had a small but exciting experience with 13 patients with SBS/CIF. This paper intends to reflect the reality of a small country with limited economic resources, where, despite a small number of patients with CIF, the clinical challenges were successfully surpassed. Nine of these 13 patients had acute mesenteric ischemia, which led to SBS. The other four patients, the SBS/CIF, result from radiation enteritis (two), extensive intestinal resection due to a mesenteric desmoid tumor, and intestinal aganglionosis. This small number of patients in HPN and the type of underlying pathology can result from some negative factors of clinical practice and some positive ones. As negatives, we can include the later diagnosis, elderly patients, and multiple comorbidities leading to the death of many patients with acute mesenteric ischemia. As positives, the advances in the treatment of some pathologic conditions, such as the biological treatment of Crohn's disease, the implementation of radiotherapy neoadjuvant and not adjuvant in locally advanced rectal cancer with reduction of radiation enteritis, and the collaboration of vascular surgery in the management of acute and chronic mesenteric ischemia, led to a decrease in the number of patients with SBS/CIF. The absence of oncologic patients with intestinal obstruction in our HPN program is probably due to the poor survival of these specific patients and the difficulty of providing the home-efficacy home care support they need. 

Although SBS/CIF is rare, its clinical management is complex, challenging, expensive, and time-consuming. A correct approach to this condition, a multidisciplinary team [14], an HPN program implementation, and open SOS phone contact are necessary.

These patients have in common the need for several surgeries of intestinal resections, and most need intensive care hospitalization. In most cases, the remnant intestine of the patients operated on due to acute mesenteric ischemia was a small amount of small bowel (10 cm to 60 cm) and the left colon. When possible, elective intestinal reconstruction was made to make HPN management easier [15]. In our series, the absence of stomas was feasible in 10 of 13 patients. Doing intestinal reconstruction seems to be a good achievement and allows for the reduction of hydroelectrolytic imbalance and cardiac and renal dysfunction.

On the other hand, HPN remains the standard of care for those patients with SBS/CIF, but the complications related to HPN are frequent and life-threatening [4]. Implementing an HPN program since the SBS/CIF patient was metabolically balanced and non-septic is difficult. We took some strategies to reduce HPN complications: implementation of a patient education and training program for HPN administration; the patient's monitoring by the same team in medical appointments and during hospitalization; the correct use of parenteral and enteral support; and the utilization of the analog of human GLP-2, whenever indicated. 

These patients require long-term and lifelong vascular access and are at considerable risk of developing central line-associated bloodstream infections. In our experience, the most frequent complication was a CVC infection. This type of infection is a frequent and severe problem. It can provoke severe sepsis, territory venous thrombosis, and even threaten patient life. In our patients, CVC infection results in multiple hospital admissions for sepsis, three patients with extensive thrombosis of venous territory, and the death of two patients. Some catheter sepsis was also related to urinary tract infection and bacterial endocarditis, worsening renal or cardiac function, or leading to renal or cardiac failure. It happened to four of our patients.

In our center, the CVC changes are realized with echography control to reduce the risk of catheter infection and thrombosis in the venous territory. Catheter-related bloodstream infections are a severe complication of HPN. Antibiotic salvage of CVCs in CRBSI is recommended; however, this is based on limited reports [16]. In our center, in cases of infection, a standardized 10- to 14-day catheter salvage treatment protocol, including antibiotic CVC locks, stopping bag perfusion, and systemic antibiotic administration, was used to salvage infected CVCs as appropriate. The CVC was not used for systemic therapy for the duration of the salvage protocol. We don’t routinely use taurolidine + 4% citrate after the nutrition bag infusion as infection prevention, although other centers do it with promising results [17]. Relatively to central venous thrombosis, a vascular surgeon with exceptional skills in the vascular access approach has resolved the problems (Figure 2).

To avoid metabolic complications, we select and prescribe the nutrition bags, monitor the patient's nutritional and health status, and try the intelligent detection of HPN side effects. Moreover, we use the enteral route whenever possible. It is essential to highlight the possible nutrient absorption via the enteral route, which contributes to the total intake depending on the patient's characteristics. This route is also critical for maintaining enterohepatic circulation, reducing the risk of parenteral nutrition-associated complications, namely hepatic failure. Specific oral nutritional supplementation can also be a strategy to mitigate parenteral intake. Oral nutritional support is necessary to avoid over-nourishing patients, but we cannot accurately quantify this. Still, we can confirm this happens because body weight is maintained with parenteral administrations that are lower than the calculated energy expenditure.

The use of individualized bags can contribute not only to the reduction of metabolic complications but also to the reduction of catheter infections and hydroelectrolytic imbalances. We have recently started using custom bags to reduce the complications of HPN. Whenever it is indicated to reduce the volume/number of HPN bags per week [18], Teduglutide is an essential aid in managing these patients [19]. In addition to reducing the number of bags, it reduces the long-term complications of HPN and significantly improves the patient's quality of life [20, 21]. This analog of human GLP-2 has been referred to as improving structural and functional intestinal adaptation following intestinal resection by decelerating rapid gastric emptying, decreasing gastric hypersecretion, increasing intestinal blood flow, and promoting intestinal growth [22]. It was approved in Portugal only in 2019. In our series, it was possible to introduce teduglutide to two patients. Although still recently introduced, this drug was well tolerated, with a reduction in the number of bags per week.

Liver failure with progression to cirrhosis is the HPN complication most people fear. The bag composition (calory, the ratio glucose/lipide, and fish oil emulsion) can interfere with liver function [23-26]. Monitoring and bag composition readjustment are essential tasks in outpatient appointments. Monitoring fluid, electrolyte, macronutrient, and micronutrient status can minimize significant organ dysfunction and metabolic complications. It should be noted that the eight patients on HPN do not show signs of liver dysfunction, with progression to cirrhosis. Hepatic cirrhosis was one of the complications that arose in one of the first patients in the series, and he died before having the opportunity to undergo the liver and intestinal transplant for which he was proposed. 

Other issues that appear during and related to HPN support include acute kidney and chronic insufficiency, cardiac insufficiency, cholelithiasis, hyperglycemia, vitamin D insufficiency, and osteoporosis, which can be resolved or controlled with the indicated measures. 

In general terms, in our center, although we have a small group of patients with various comorbidities, HPN has performed well with considerable patient survival combined with a reasonable quality of life. In our series, patients have an average of 173 months of HPN. This outcome is identical to that of other centers with enormous experience [27-30]. This result was and is only possible through the joint action of a multidisciplinary group, including general surgery, vascular surgery, nutritionists, nursing, pharmacy and nephrology, cardiology, and anesthesiology, to effectively treat patients with SBS/CIF.

## Conclusions

Home parenteral nutrition remains the gold standard in SBS/CIF treatment, though teduglutide may reduce HPN requirements and complications while improving quality of life. Despite the small number of patients, the results of this study are comparable to those of larger centers. The commitment and interest of professionals involved in SBS/CIF at Centro Universitário de Santo António was critical to achieving these outcomes. A multidisciplinary healthcare team for HPN support can be critical to the patients' survival and quality of life.
